# Organizing the Global Diversity of Microviruses

**DOI:** 10.1128/mbio.00588-22

**Published:** 2022-05-02

**Authors:** Paul C. Kirchberger, Zachary A. Martinez, Howard Ochman

**Affiliations:** a Department of Molecular Biosciences, University of Texas at Austin, Austin, Texas, USA; University of Pittsburgh

**Keywords:** *Microviridae*, single-stranded DNA viruses, taxonomy, metagenomes

## Abstract

Microviruses encompass an astonishing array of small, single-stranded DNA phages that, due to the surge in metagenomic surveys, are now known to be prevalent in most environments. Current taxonomy concedes the considerable diversity within this lineage to a single family (the *Microviridae*), which has rendered it difficult to adequately and accurately assess the amount of variation that actually exists within this group. We amassed and curated the largest collection of microviral genomes to date and, through a combination of protein-sharing networks and phylogenetic analysis, discovered at least three meaningful taxonomic levels between the current ranks of family and genus. When considering more than 13,000 microviral genomes from recognized lineages and as-yet-unclassified microviruses in metagenomic samples, microviral diversity is better understood by elevating microviruses to the level of an order that consists of three suborders and at least 19 putative families, each with their respective subfamilies. These revisions enable fine-scale assessment of microviral dynamics: for example, in the human gut, there are considerable differences in the abundances of microviral families both between urban and rural populations and in individuals over time. In addition, our analysis of genome contents and gene exchange shows that microviral families carry no recognizable accessory metabolic genes and rarely, if ever, engage in horizontal gene transfer across microviral families or with their bacterial hosts. These insights bring microviral taxonomy in line with current developments in the taxonomy of other phages and increase the understanding of microvirus biology.

## INTRODUCTION

The vast majority of viruses in the human gut are single-stranded DNA (ssDNA) or double-stranded DNA (dsDNA) phages (1). dsDNA phages, as exemplified by T4, T7, and Lambda, have been systematically classified into 14 families, 73 subfamilies, and 927 described genera. In contrast, the most abundant group of ssDNA phages, the *Microviridae*, consists of only a single family, split into two subfamilies and seven described genera (2). The low number of microviral taxa belies their broad distribution and diversity, with metagenomically assembled genomes (MAGs) recovered from a very wide range of environments (3, 4). Based on the phylogenetic breadth of their bacterial hosts, the origin of these small, tailless microviruses is hypothesized to trace back billions of years, perhaps before the last universal ancestor of bacteria (5). Given this ample time to evolve, the current classification of *Microviridae* mostly reflects difficulties in isolating and describing these viruses rather than the diversity that is known to exist.

Prominent among the characterized *Microviridae* are the environmentally rare but intensely studied *Bullavirinae*, as represented by the iconic *phi*X174 (6), and the abundant *Gokushovirinae*, with lytic isolates in Chlamydia, Spiroplasma, and Bdellovibrio and temperate ones in *Enterobacteriaceae* (7). In the last decade, metagenomic studies have also uncovered a vast amount of unclassified diversity within the *Gokushovirinae*, *Bullavirinae*, and other microviruses. Microviral sequences detected in the genomes of *Bacteroidetes* from the human gut were assigned to a new putative subfamily named Alpavirinae (8), and another subfamily, the Pichovirinae, known exclusively from MAGs from the human gut, has been proposed (9). Divergent microviral MAGs have also been recovered from dragonflies (Group D) (10), peatland water and soil (Aravirinae and Stokavirinae) (11), the guts of marine tunicates (*Ciona* gut microphage/CGM) (12), and termites (Sukshmavirinae) (13). Renewed efforts at isolating microviruses have recovered additional lysogenic and lytic microviruses in *Alphaproteobacteria* (Amoyvirinae) (14–16), and a recent survey of mammalian gut metagenomes recommended the establishment of 10 additional subfamilies (17). Overall, thousands of microvirus MAGs have been assembled and a substantial number of microviral prophages have been detected in bacterial genomes (18). Collectively, these studies have elevated the number of actual or candidate microviral subfamilies from the original 2 to 20, exposing a diversity that has rapidly outpaced the precise delineation of new taxa: notably, only the original *Bullavirinae* and *Gokushovirinae* are taxa accepted by the International Committee on Taxonomy of Viruses.

In this study, we analyze a comprehensive set of microviral genomes, offer a robust taxonomy, and provide insights into the diversity, distribution, and host range of this large group of small viruses. In addition, we provide a curated data set of annotated microviral genomes that are taxonomically assigned by a computational pipeline (Microvirus Organization Pipeline Using Protein sharing [MOP-UP], available at https://github.com/martinez-zacharya/MOP-UP). Like vConTACT 2 (19), this pipeline creates networks of related genomes based on the amino acid identity of shared proteins, but it has been streamlined for microviral genomes.

## RESULTS

### Microvirus diversity remains undersampled.

**FIG 1:**
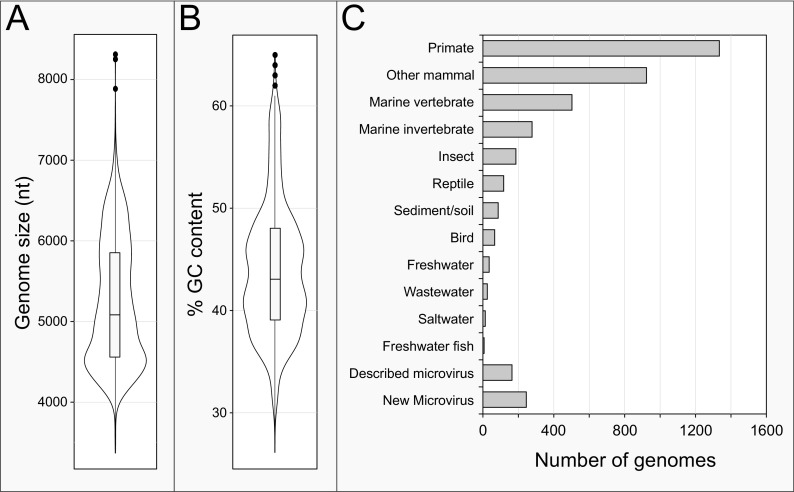
Properties and sample origins of *Microviridae* genomes. (A) Violin and box-and-whisker plots of size distribution of microviral genomes. (B) Violin and box-and-whisker plots of GC contents of microviral genomes (same data set as in panel A). Box-and-whisker plots show median values, 25th and 75th percentiles, and 1.5 interquartile ranges, as well as outlier data points. (C) Histogram showing the sources of samples from which microviral genomes were detected/isolated. The bar labeled “Described microvirus” denotes genomes that can be assigned to official microviral genera, and the bar labeled “New microvirus” denotes genomes that can be assigned to bacterial hosts either as prophages or via CRISPR arrays.

To achieve a comprehensive understanding of the diversity within the *Microviridae*, we assembled a data set of 4,077 complete, manually curated microviral genomes consisting of published isolate sequences, metagenome-assembled genomes (MAGs), and prophage sequences that we discovered through iterative hidden Markov model (HMM) searches for microviral major capsid proteins (Table S1 in the supplemental material). The median genome size in this data set is 5,078 nucleotides (nt), the largest being 8.3 kb (MG945451, a circular MAG isolated from yak feces) and the smallest 3.5 kb (MH617603, a MAG from minnow tissue) (Fig. 1A). The median GC content is 43%, but it ranges from 26% GC in a microviral circular genetic element of a Chlamydia abortus genome (FPMJ01000014) to 65% GC in an Apis mellifera-associated MAG (MH992159) (Fig. 1B). After dereplication of the data set based on the sharing of conserved proteins at ≥50% amino acid identity (AAI), the microviral genomes form 1,691 subgroups roughly corresponding to the taxonomic rank of genus. Of these, 1,152 subgroups that together represent 28% of all genomes contain only a single genome, indicating distinct undersampling at this taxonomic level.

The majority of microviral genomes in our data set originate from the viromes of humans and other primates, followed by nonprimate mammals and marine organisms, representing the bias toward sampling these environments (Fig. 1C). Approximately 11% of the genomes could be assigned to bacterial hosts as isolates or via their presence as integrated or circular genetic elements in bacterial genomes. Additionally, CRISPR-based predictions assigned bacterial hosts to ~20% of the data set, and only 5 of the 216 predictions for phages with confirmed hosts were incorrect. Over 40% of the microviruses that can be linked to hosts are members of previously described microviral genera, such as *phi*X174 microvirus and *Enterogokushovirus*. However, several hundred phages that were definitively assigned to specific bacterial hosts represent novel microviruses that have yet to be isolated (Fig. 1C). Overall, the hosts to which microviruses were assigned span 17 bacterial phyla, 28 classes, 53 orders, 79 families, and 135 genera (Table S1), with most hosts corresponding to phyla previously reported to be infected by *Microviridae* (e.g., *Proteobacteria* and *Bacteroidetes*).

Individual microviral genomes were associated with *Nitrospirae*, *Cyanobacteria*, *Actinobacteria*, *Spirochaetes*, the candidate phyla “*Candidatus* Melainabacteria” and “*Candidatus* Patescibacteria,” and the archaeal phylum *Methanomicrobia*, but upon close inspection, each of these genomes is represented by a single contig in fragmented, metagenomically assembled bacterial genomes, a notoriously error-prone process. Similarly, the few microviruses ascribed to Gram-positive bacteria are mostly present in metagenomically binned sequences, not in complete genomes. We did, however, detect complete microviral prophages in the genomes of Erysipelatoclostridium and Mammaliicoccus sciuri isolates (phylum *Firmicutes*), which represent the first cases of microviruses reported in Gram-positive bacteria.

### Microviral diversity can be partitioned into three putative suborders and 19 families.

**FIG 2:**
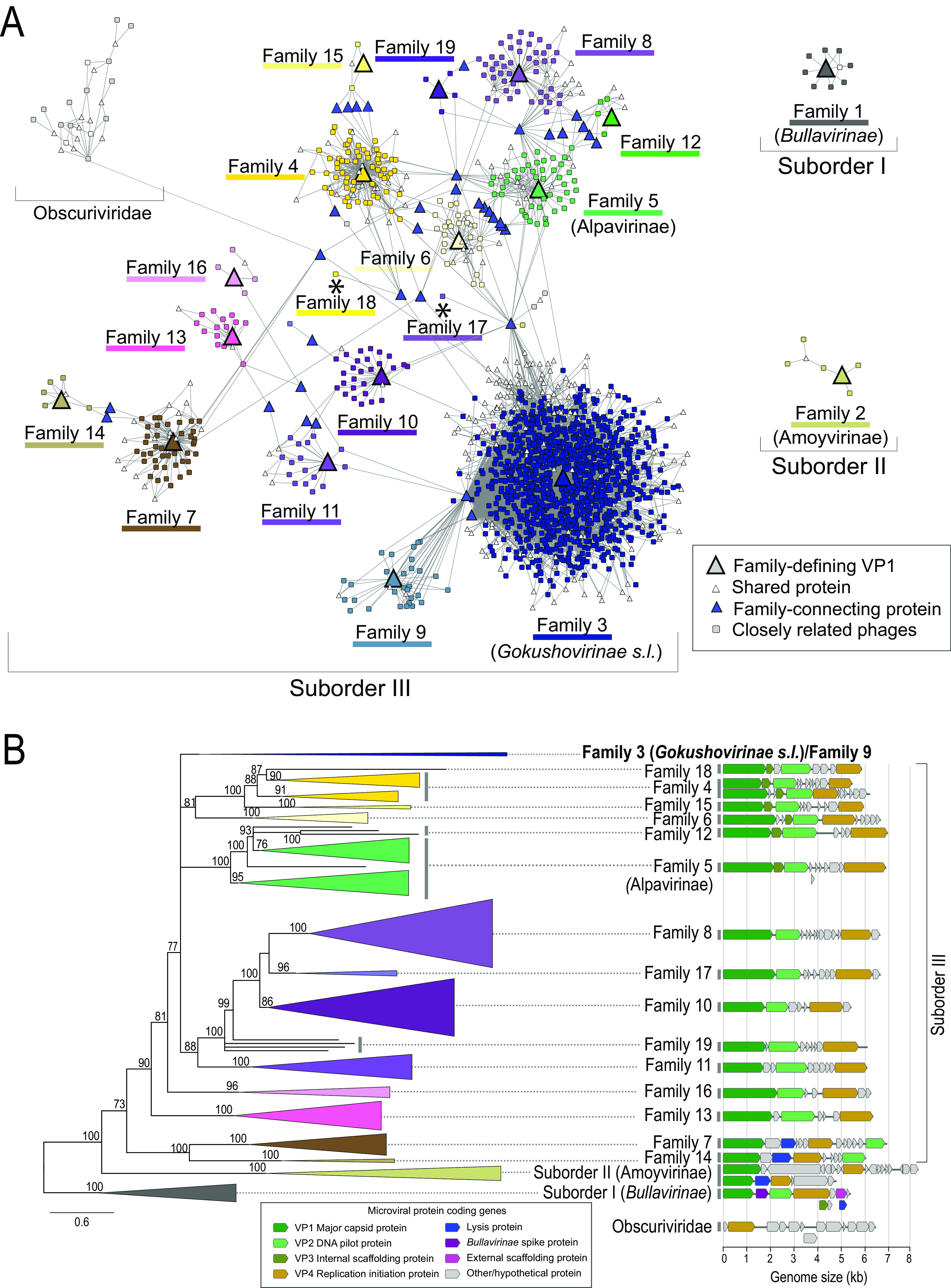
Diversity, relationships, and genome contents of microviruses. (A) Bipartite protein-sharing network and family assignments of microviruses. Groups of related phages (rectangles) are connected by groups of shared proteins (triangles) at ≥30% amino acid identity. Each phage family (colored) is defined by a shared VP1 major capsid protein at ≥30% amino acid identity (AAI), denoted by large colored triangles. Blue triangles represent proteins shared between members of different putative families at ≥30% AAI. Families 17 and 18 (labeled with asterisks) are each composed of closely related genomes that are not connected though a shared VP1. Singleton genomes not connected to a family-defining VP1 protein at ≥30% AAI are not depicted. (B) Phylogenetic tree and genome contents of microviruses. Maximum-likelihood tree based on VP1 and VP4 proteins. Families are depicted as elongated triangles that retain maximum branch length and are colored as in panel A. With the exception of Family 3/Family 9, sizes of clades represent the overall diversity after removal of redundant branches. Numbers on branches indicate transfer bootstrap estimates (61), with branches of <70 collapsed. Scale bar indicates amino acid substitutions per site. For each family, representative genomes are depicted linearly starting with VP1 (dark green). Note that overprinted open reading frames, with the exception of those reported in Family 1 (*Bullavirinae*), are not indicated. *s.l.*, *sensu lato*.

To establish higher-order relationships among microviruses, we constructed bipartite protein-sharing networks, in which groups of closely related genomes are connected to more distantly related genomes through the proteins shared by both. Applying a threshold of 30% AAI over 80% of protein length results in clusters of genomes at 17 centrally connected VP1 major capsid proteins; VP1 is the hallmark phylogenetic marker of the *Microviridae* (Fig. 2A). We consider these 17 clusters, together with two additional groups consisting of more than 5 genomes, as corresponding to a total of 19 putative families of microviruses (a tentative taxonomic rank that allows amendment and refinements at higher and lower levels, noting that in multiple instances, the suffixes of -*idae* and -*inae* are technically incorrect but retained to avoid confusion). Another cluster (labeled Obscuriviridae in accordance with Bartlau et al. [20] in Fig. 2) represents ssDNA phages that previously were classified as microviruses (21) but contain no recognizable microvirus-specific proteins. The predicted structure of their putative capsid proteins most resembles that of the family *Finnlakeviridae* (22, 23) (Dali Z score [24] of 15.3 for PDB accession number 5OAC), and they should not be considered members of the *Microviridae*.

To confirm the integrity of the 19 remaining microviral families, we performed phylogenetic analysis of a concatenated alignment of VP1 and VP4 proteins (the major capsid protein and replication initiation protein, respectively) (Fig. 2B). This phylogeny shows that some families are nested within larger families (e.g., Family 18 emerges from within from Family 4) and that the four lineages within Family 19, although each other’s closest relatives, do not form a monophyletic group. Overall, however, the phylogenetic clades are consistent with the network-based clusters, and the majority of families recognized by protein-sharing networks are monophyletic.

Based on the partitioning in the protein-sharing network and phylogenetic analysis (Fig. 2), the 19 microviral families assort into three major divisions that we tentatively term suborders and that encompass over 99% of known microviral diversity (see Discussion and reference 25), as follows.

**FIG 3:**
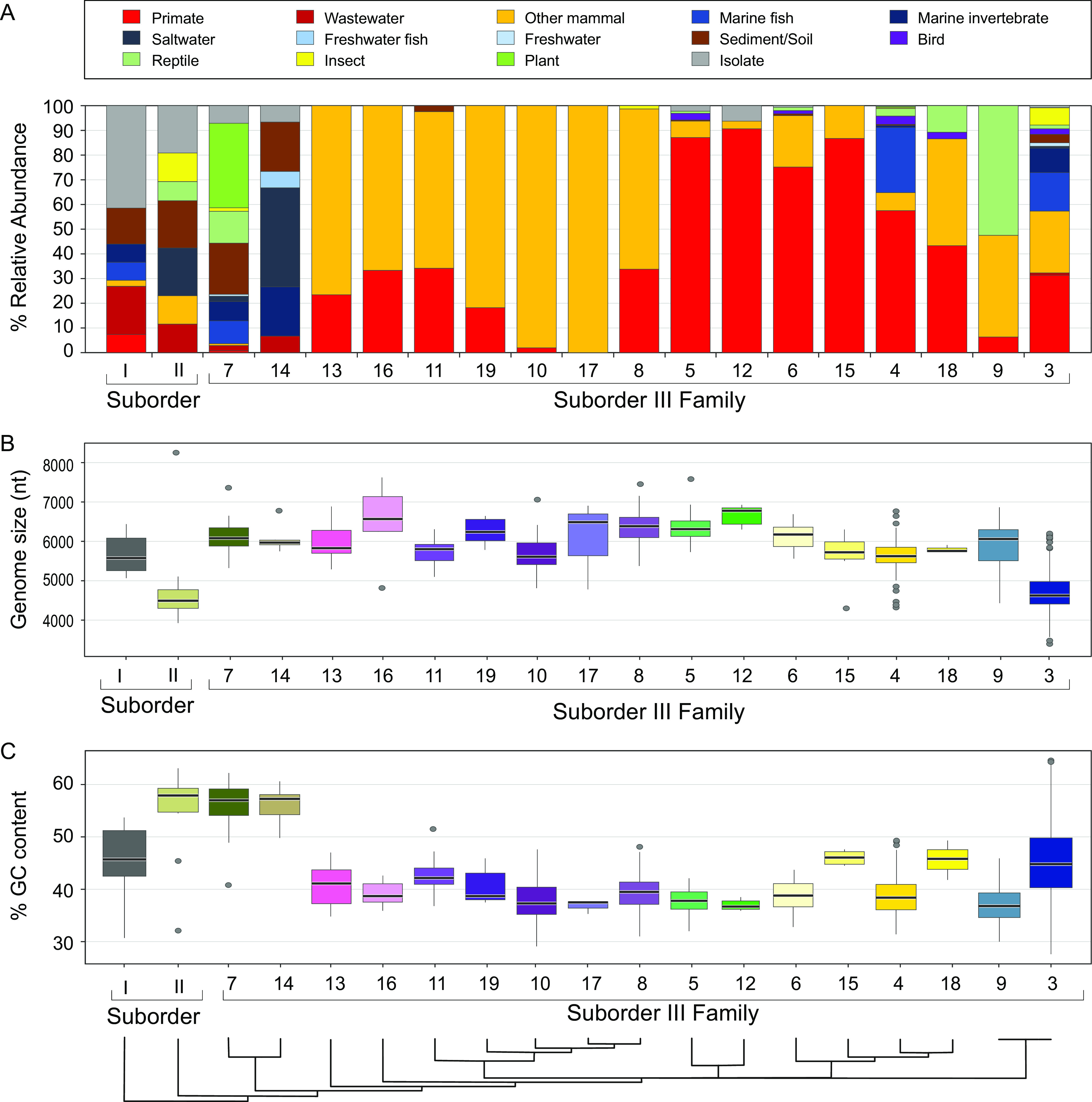
Sample distribution and genomic features of putative microviral families. In all panels, families are ordered from left to right according to phylogeny shown in Fig. 2B, recapitulated at the bottom of the figure. (A) Origin of genomes from each microviral family. (B) Genome size distribution of microviral families. Several families contain outliers in genome size due to putatively truncated MAGs containing all hallmark genes and/or to oversized prophages with undefined insertion boundaries. (C) GC contents of microviral families. (B and C) Box-and-whisker plots are color coded as in Fig. 2 and show median values, 25th and 75th percentiles, 1.5 interquartile ranges, and outlier data points; individual data points are derived from single genomes randomly chosen to represent their respective genera.

(i) Suborder I consists of Family 1 and includes all described genera of the subfamily *Bullavirinae*, Klebsiella prophages, and several MAGs associated with the closely related proposed Pequenovirus taxon. Hallmarks of this suborder that are missing from other suborders include the presence of a lipopolysaccharide (LPS)-binding spike protein and an external scaffolding protein involved in capsid assembly (Fig. 2B). Suborder I is also the only taxon with a considerable number of isolates in the form of *phi*X174-like phages (Fig. 3A).

(ii) Suborder II encompasses Family 2, as well as phages infecting Ruegeria (previously grouped into the subfamily Amoyvirinae [14]). Suborder II phages have undergone little study and, thus, can only be defined by the lack of a recognizable VP 2 DNA pilot protein. Members of the suborder have small genomes, except for the divergent Liberibacter prophages, whose genome size is almost twice that of other members of this suborder (although note that its unusual genomic structure could also be indicative of insertions or genomic rearrangements, as shown in Fig. 3B). Notably, almost all members of Suborder II have distinctly high GC contents (Fig. 3C).

(iii) Suborder III subsumes Families 3 through 19, most of which derive members predominantly from the guts of primates and other mammals (Fig. 3A). Family 3, within Suborder III, is the largest in terms of its numbers of genomes and genera (2,650 genomes in 1,139 genera) and encompasses multiple taxa that were previously referred to as subfamilies (including *Gokushovirinae*, Pichovirinae, Stokavirinae, Aravirinae, Sukshmavirinae, Group D, and Parabacteroides prophages, although some Parabacteroides prophages also exist in Family 6). Also within Suborder III, Family 5 contains the Alpavirinae, another previously described subfamily that mostly infects Bacteroides and Prevotella. Families 7 and 14 contain high-GC-content MAGs (Fig. 3C) that were first described as a subfamily of *Ciona* gut microphages (CGM), plus lytic and temperate phages infecting marine *Rhodobacteraceae* and soil/plant-associated *Hyphomicrobiaceae*. For the most part, the gene order of conserved, nonaccessory genes of phages within Suborder III is maintained: genomes are almost exclusively ordered VP1–VP2–VP4 (circular genomes are arbitrarily considered to begin with VP1 at the linearized 5′ end), with variation observed in Families 7 and 14 (VP1–peptidase/amidase–VP4–VP2) and in the location of VP3 (internal scaffolding protein, an equivalent of which exists in Family 1) (Fig. 2B). Family 3 is exceptional with respect to gene order: here, all six possible variations on the conserved gene order are observed (Fig. 2B). Structurally resolved isolates of Suborder III (more specifically, the *Gokushovirinae*) sport a mushroom-like protrusion on their viral capsid, formed by hypervariable loop regions in their VP1 proteins (9, 26). Such hypervariable regions in the VP1 protein can be found in almost all families of the suborder, indicating that gokushovirus-like protrusions might be a defining feature of Suborder III.

### Subfamilies populating putative microviral Family 3.

**FIG 4:**
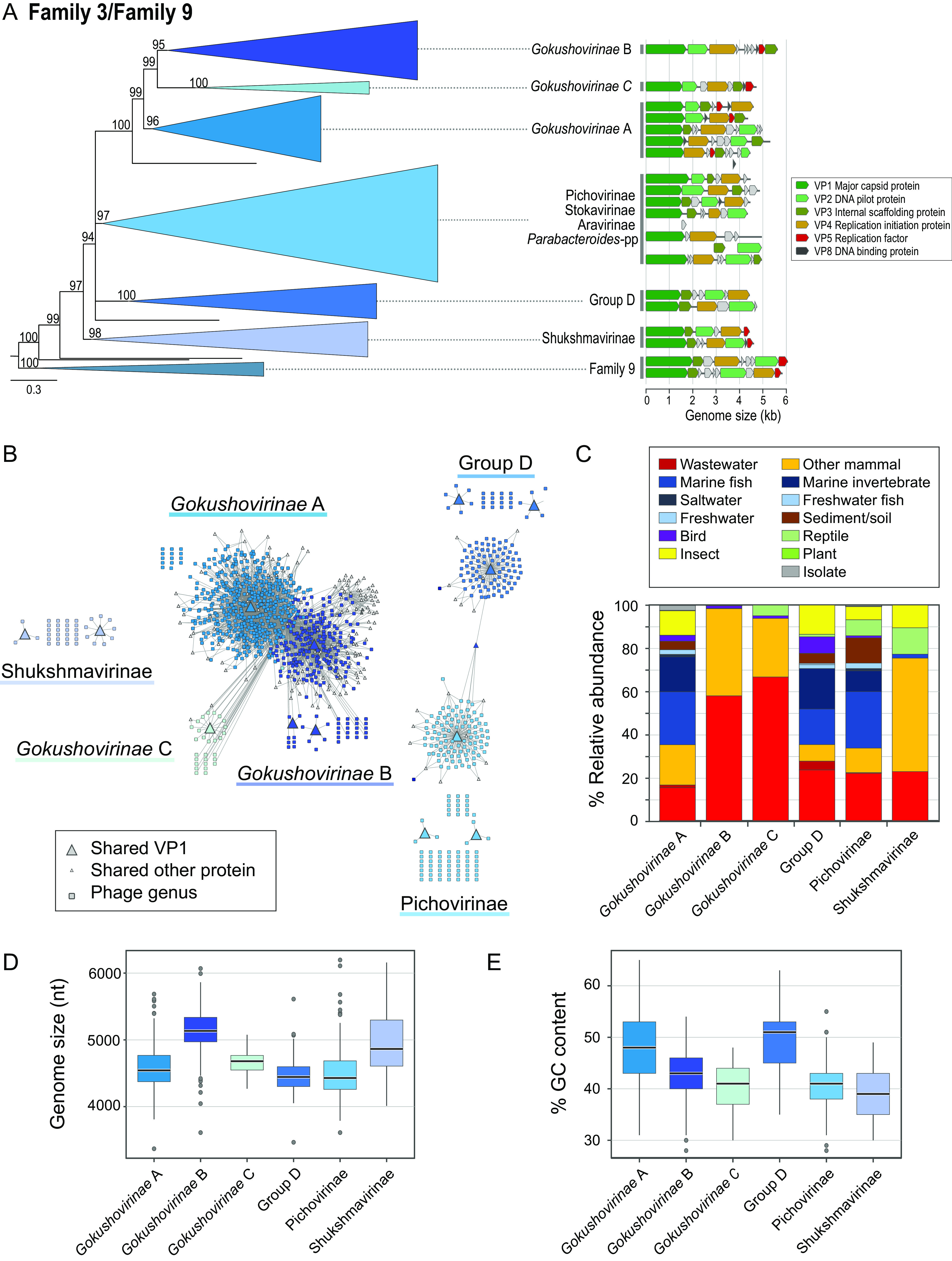
Phylogenetic diversity, sample origins, and genomic features of Family 3/Family 9 microviruses (*Gokushovirinae sensu lato*). (A) Phylogenetic tree of Families 3 and 9. Maximum-likelihood tree based on VP1 and VP4 proteins. Subfamilies are depicted as elongated triangles that retain maximum branch length. Sizes of clades represent the overall diversity after removal of redundant branches. Numbers on branches indicate transfer bootstrap estimates (61), with branches of <70 collapsed. Scale bar indicates amino acid substitutions per site. Tree was rooted with Family 9. For each subfamily, representative genomes are depicted linearly starting with VP1 (dark green). (B) Bipartite protein-sharing network of Family 3. Phage genera are depicted as rectangles, and proteins shared between genera at ≥50% amino acid identity as triangles. Colors correspond to phylogenetically defined subfamilies as in panel A and can encompass multiple VP1 clusters (large triangles). (C) Origins of genomes from each Family 3 subfamily. (D) Genome size distribution of Family 3 subfamilies. Several subfamilies contain outliers in genome size due to putatively truncated MAGs containing all hallmark genes and/or to oversized prophages with undefined insertion boundaries. (E) GC content distribution of Family 3 subfamilies. (D and E) Box-and-whisker plots are color coded according to phylogenetically defined subfamilies in panel A and show median values, 25th and 75th percentiles, 1.5 interquartile ranges, and outlier data points. (B and C) Individual data points are derived from single genomes randomly chosen to represent their respective genera.

The largest microviral family, Family 3, contains seven previously proposed subfamilies: the officially accepted *Gokushovirinae*, the *Parabacteroides* prophages, and five taxa (the Sukshmavirinae, Aravirinae, Pichovirinae, Stokavirinae, and Group D phages) known only from MAGs. We investigated the structure within Family 3 by phylogenetic tree construction and by applying a more stringent threshold (≥50% AAI) for protein-sharing networks. At this threshold, several small clusters and singletons formed in the protein-sharing network are closely related to or contained within larger clades from the phylogenetic analysis, allowing these lineages to be subsumed into the established subfamilies (Fig. 4A and B). From these analyses, the *Gokushovirinae* (which, in aggregate, amount to roughly half of the genomes and 70% of network-defined genera) separate into three clades, which we term *Gokushovirinae* A, B, and C. Among officially recognized genera, *Gokushovirinae* A includes the Bdellovibrio*-*, Chlamydia*-* and Enterobacteria-infecting gokushoviruses, *Gokushovirinae* B includes the described lineage infecting Spiroplasma, and Gokushovirinae C includes only MAGs. Genome organization is highly variable within *Gokushovirinae* A, whose genomes have been recovered from a variety of environments, whereas *Gokushovirinae* B genomes are larger and more uniform and are almost exclusively associated with mammals (Fig. 4B to D).

Two additional, well-supported phylogenetic clades encompass multiple network clusters and correspond to the previously proposed Group D (genomes of which trend toward higher GC content, as seen by the results shown in Fig. 4E) and Sukshmavirinae subfamilies (Fig. 4A and B). Furthermore, a single large phylogenetic clade encompasses multiple clusters in the ≥50% AAI network and includes the Aravirinae, Pichovirinae, Stokavirinae, and Parabacteroides prophages (Fig. 4A and B). Within this specific clade, weak bootstrap support and disagreements between phylogeny and network clusters (genomes on long branches within a clade form unconnected singletons or new clusters in the network) preclude assignment of genomes to those five named taxa, and we subsume them under the name Pichovirinae. Overall, there are multiple divisions within Family 3 that could be considered subfamilies, which stands in contrast with the multiple families and suborders that were previously ranked as subfamilies. As such, previous designations of microviral “subfamilies” reside at drastically different taxonomic levels.

### Microviruses have limited accessory gene repertoires and are genetically isolated from the larger microbial pangenome.

Despite the diversity and number of microviruses included in our analyses, we found no evidence of accessory metabolic genes in any genome. However, several microviruses possess accessory methyltransferases with a putative role in escaping host restriction, as well as genes likely to be involved in host cell lysis, such as peptidase genes (Fig. 2B, Fig. S1) (9). Lysis-associated accessory genes are conserved in genomic locations between VP1 and VP4 in phages of Families 2, 7, and 14 (Fig. 3A). Phages in other families also occasionally contain accessory genes with the aforementioned functions at a variety of genomic locations (Fig. S1). Of note is that our analysis omits overprinted genes, which are known to be present in at least the *Bullavirinae* of Family 1 but cannot be verified based solely on computational methods.

In some instances, accessory proteins connect individual members of different microviral families in the protein-sharing network (Fig. 2A, Table S2). Most connections are created by small (65 amino acids [aa] on average) proteins/peptides, of which only a few can readily be assigned a function. Such connections could be spurious, especially in cases of small peptides, but they can also derive from shared ancestry, recombination between microviruses, or separate acquisition from nonmicroviral sources. For example, a hypothetical protein of ~200 aa in size links distantly related Families 3, 4, and 7 and the nonmicroviral “Obscuriviridae.” The proteins belonging to different microvirus families share 30 to 40% identity with each other but also with numerous bacteria and dsDNA phages; as such, they likely represent independent acquisition events. In another instance, a peptidase protein links Suborder II with Families 3 to 6, 8, and 10 of Suborder III. Here, two MAGs from different families (MG945328 and MG945336) display 21% AAI in their VP1 protein but 74% AAI in their shared peptidase. Upon closer inspection, this protein is encoded in a 700 nt region of elevated nucleotide identity (72%, versus 38% for the rest of the genome), evidence for occasional recombination events between distantly related microviruses (Fig. S2).

Nonaccessory microviral proteins (denoted with a VP prefix, e.g., the major capsid protein VP1) likely share common ancestry, but only in a few instances do they connect families or even subfamilies at the threshold of >30% AAI or >50% AAI, respectively (Fig. 2A and 4B). For example, a VP4 protein cluster connects Family 11 to Families 3/9, and a DNA binding protein (VP8) connects some Pichovirinae with a member of Group D phages. Other connections, between Families 4 and 15, 3 and 9, or among gokushoviral subfamilies (which are connected via a conserved VP8 protein), most likely denote common ancestry between closely related (sub)families. Overall, the lack of connectivity between microviral families is indicative of both genetic isolation and rapid gene content and sequence evolution among families of microviruses.

### Rapid classification of thousands of new microviruses.

New metagenomic sequencing projects are constantly yielding unprecedented amounts of novel viral sequences, far exceeding our curated set of microviral genomes in number. To simplify investigation of microvirus diversity from such new sequencing projects, we formulated our methods into a pipeline—Microvirus Organization Pipeline Using Protein sharing (MOP-UP). MOP-UP expedites the classification and discovery of novel microviruses by providing a protein-sharing-network graph that connects new genomes to the curated set of *Microviridae*, thereby sorting them into the taxonomic groups described above.

We first analyzed 14,350 contigs larger than 4,000 nt from a wastewater metagenomic data set, specifically enriched for small, circular DNA elements (27). The output from MOP-UP produces a clear separation of microviral genomes from most other sequences at a 30% AAI cutoff (Fig. S3). (Note that a large cluster of plasmids is connected to the *Microviridae* through homologous VP4 replication initiation proteins). Of the sequences derived from this data set, 3,871 correspond to *Microviridae*, and almost all can be assigned to major families through association with VP1 proteins encoded by established groups of microviral genomes.

We further investigated microvirus sequences from recent large-scale catalogs of human gut phages—the Cenote Human Virome Database (3) and the Metagenomic Gut Virus Database (28)—as well as microviruses from a global ocean virome data set (29) and three additional data sets from recent microvirus-related publications (30–32). Together with the aforementioned wastewater data set, we amassed 9,198 new microvirus genomes, more than twice the number of our original genomes (Table S3).

**FIG 5:**
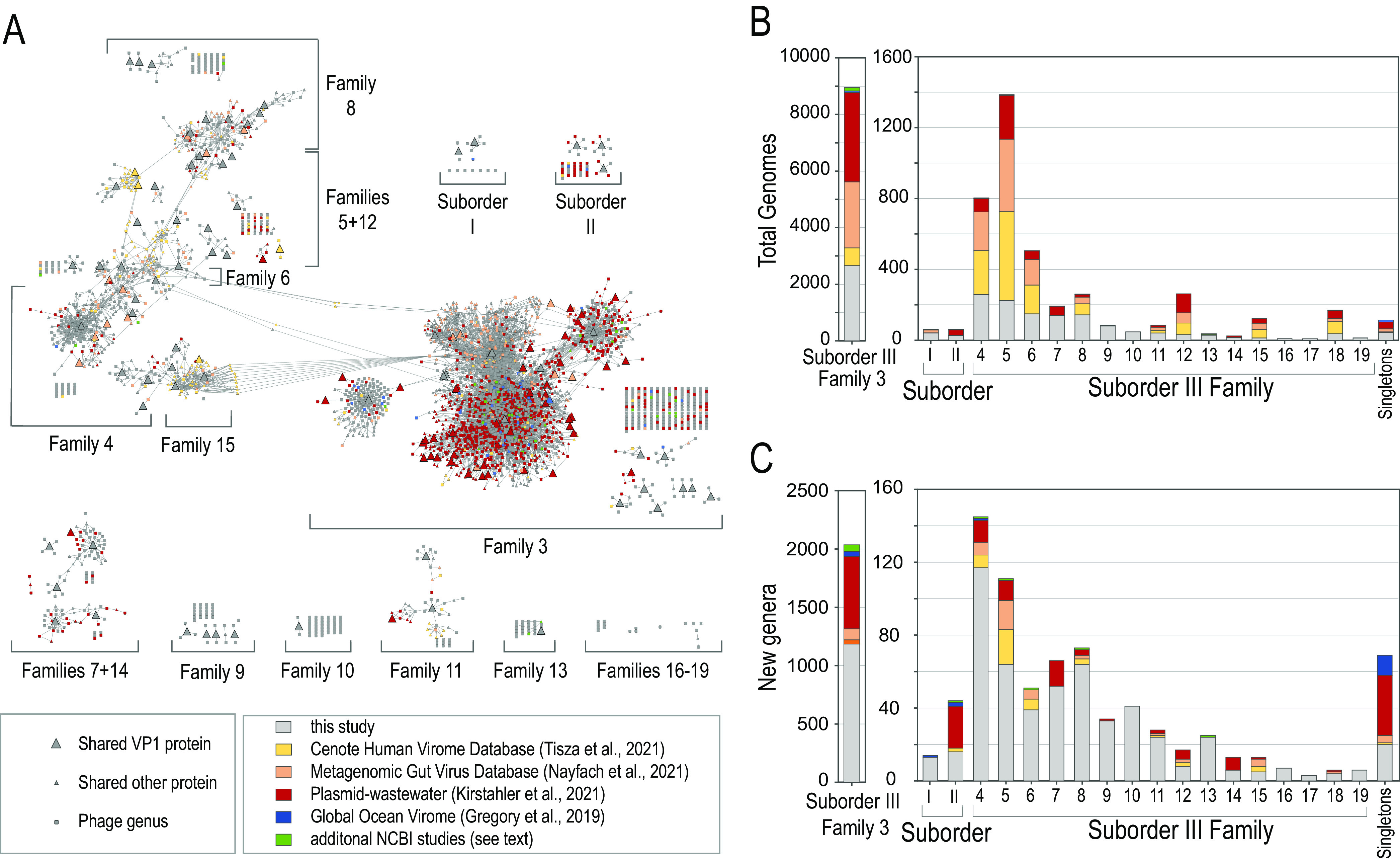
Taxonomic assignment of thousands of additional microviral genomes. (A) Bipartite protein-sharing network of microviral families showing the database source of phages. Phage genera are depicted as rectangles, and proteins shared between genera at ≥50% amino acid identity as triangles. Genera are color coded by database source, either gray when already represented in the MOP-UP database used in this study or according to the source that contributed the largest number of phages to the respective genus (see the key). Genera unconnected to any family-defining VP1 protein at ≥30% AAI are not depicted in panel A but are indicated as Singletons in panels B and C. (B) Contributions of various microvirus data sets to total numbers of genomes. (C) Novel genera discovered in additional microvirus data sets. Genera are considered novel when they do not contain phages from the MOP-UP database (this study). (B and C) Bars are colored according to database source (see the key in panel A).

Over 99% of genomes in these additional data sets are members of the new families defined in this study (Fig. 5A): the majority are assigned to Family 3, followed by other families abundant in the human gut (e.g., Families 4, 5, and 6), with only Families 10, 16, and 17 not represented. However, we detected 322 new genus-level groups consisting of least two genomes, with only 17% of those new genera present in two or more data sets. Together with 730 new genus level groups with just one genome, this represents a considerable increase in the number of genera in Families 2, 3, and 5 and implies a tremendous amount of genus-level microviral diversity. The number of singleton genera not corresponding to a family, although constituting less than 1% of genomes overall, more than doubled compared to the number in the original data set (Fig. 5B and C). Despite the broad expansion of the data set, none of the genomes produces VP1 clusters indicative of new microviral families beyond those circumscribed with the original data set (Fig. 5A). Therefore, although microviral diversity remains unsampled, evidence from a vastly expanded data set demonstrates that the majority of microviral genomes can be assigned to one of the 19 families we describe.

### Abundances and distributions of microviral taxa.

**FIG 6:**
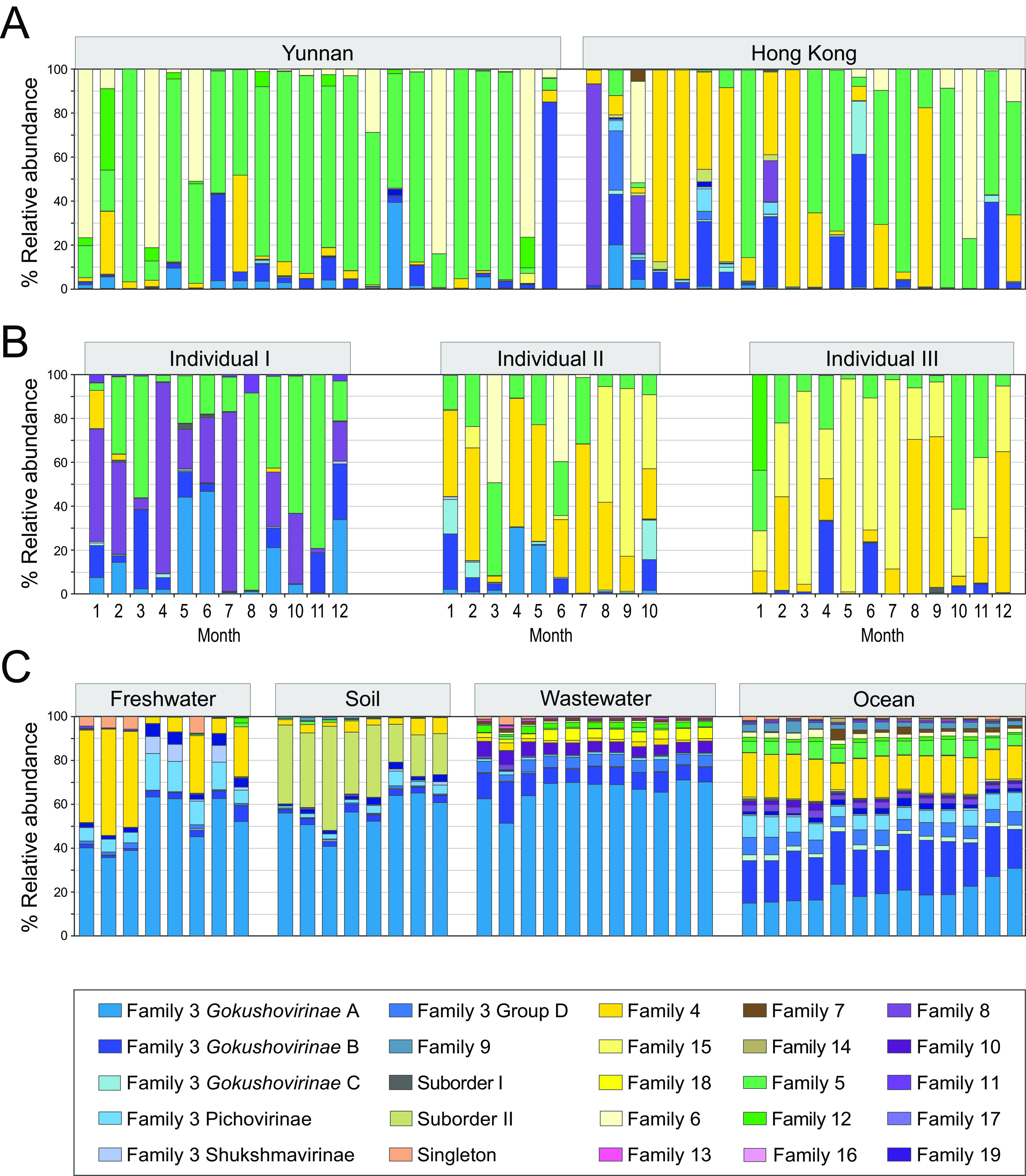
Temporal and environmental variation of microviral suborders, families, and subfamilies. (A) Abundances of microviral taxa in the viromes of individuals from Yunnan (rural areas) and Hong Kong (urban area). (B) Monthly time series of microviral taxa in the gut viromes of three individuals in Ireland. (C) Abundances of microviral taxa of metagenomes from freshwater, soil, wastewater, and ocean environments. *y* axes denote relative abundances of reads mapping onto genomes of different microviral families at ≥50% nucleotide identity.

To assess the environmental abundances of microviral families and (for Family 3) subfamilies, we mapped sequencing reads from several large-scale metagenomic studies to the genomes in our curated genome database (Fig. 6). We first investigated a small subsample of microvirus-dominated human gut viromes from rural and urban populations in mainland China and Hong Kong (33). While Family 3 gokushoviruses from mammalian guts comprise the majority of our genomic data set, the human gut contains few members of this microviral family. Instead, Families 5 and 6 are prevalent in rural gut samples (from Yunnan) and Family 4 in the urban samples (from Hong Kong) (Fig. 6A). Time series data from three urban-dwelling individuals in Ireland (3) show similar results, with Family 3 again representing only a minor component of the gut microvirome (Fig. 6B). Additionally, these longitudinal data demonstrate considerable changes in phage composition between monthly time points. For example, Family 8 phages are the dominant *Microviridae* at the beginning of sampling in Individual I but are essentially absent from Individuals II and III, where phages of closely related Families 4 or 15, respectively, make up most *Microviridae*. In the second half of the sampling period for Individual I, there is an expansion of Family 5 phages, and Family 3 (in particular *Gokushovirinae* A and B) phages become the most abundant at the end of sampling.

In contrast to the human virome, environmental samples (ocean, freshwater, soil, and wastewater) are usually dominated by Family 3 phages belonging to *Gokushovirinae* A (Fig. 6C), which correlates with the detection of MAGs from this group in a wide number of environments (Fig. 4B). However, reads mapping to *Gokushovirinae* B are about as abundant as reads in *Gokushovirinae* A in ocean metagenomes, despite previously assembled MAGs of this subfamily almost exclusively deriving from mammalian guts (Fig. 4B). Furthermore, the well-studied *Bullavirinae* of Suborder I rarely constitute even 1% of microviral reads, whereas Suborder II (Amoyvirinae) is occasionally found in the human gut but stably exists in soil environments, in accordance with their soil-dwelling *Rhizobiaceae* hosts. Overall, human gut microbiomes are dominated by different families of microviruses than other environments, with *phi*X-like phages found almost nowhere.

## DISCUSSION

Microviruses are the most widely distributed single-stranded DNA viruses on the planet but are currently classified as a single family in the viral kingdom *Sangervirae* (34). When abiding by this one-family classification, there are still at least three meaningful taxonomic levels between the ranks of family and genus. As such, the present taxonomic position creates a challenging situation in which there is insufficient room for the separation of taxa, making “subfamily” the default designation for lineages of very different levels of divergence. Therefore, even considering only the currently known lineages, the taxonomic rank of family can hardly contain the diversity of microviruses.

Beginning at the highest taxonomic level, we demonstrate that the subfamily of marine ssDNA phages first identified by Holmfeldt et al. (21) are only superficially related to the *Microviridae*. These Cellulophaga-infecting viruses were initially classified as *Microviridae* as a consequence of their ssDNA genome, icosahedral capsid, and possession of a VP4 homolog. However, apart from a broadly distributed replication initiation protein (VP4) (35), they encode no other core proteins resembling those of microviruses; in particular, they lack the hallmark VP1 major capsid protein based on which all *Microviridae* are classified. As a distinct and separate lineage of uncertain taxonomic relationships (possibly related to the *Finnlakeviridae*, based on capsid protein structure), these Cellulophaga-infecting phages should be considered separate from the *Microviridae*, and the family name “Obscuriviridae” has recently been proposed (20).

Within the true *Microviridae*, there are three clear divisions that all possess a recognizable microviral major capsid protein: the *phi*X-like *Bullavirinae*, the suggested Amoyvirinae, and a group composed primarily of *Gokushovirinae* but containing over 95% of all microviral genera and assorting into 17 clusters, which we refer to as putative families. As exemplified by putative Family 3 (the *Gokushovirinae sensu lato*), these themselves can be subdivided into even more groups previously described as subfamilies and thousands of putative genera. Even within the confines of the officially recognized subfamily of the *Gokushovirinae* in putative Family 3, a deep phylogenetic split separates mammal-associated lineages with comparatively large, uniform genomes (*Gokushovirinae* B) from the smaller, more diverse gokushoviruses that are also abundantly found in other environments (*Gokushovirinae* A). In light of these results, and the recent elevation of other viral families to higher taxonomic levels (6, 36, 37), it is fitting to raise the microviruses to the rank of order, forming three suborders (Bullavirineae, Amoyvirineae, and Gokushovirineae), each with their respective families, subfamilies, and genera (see Table S4 for an overview). Given the long history of microvirus research and use of the taxon name *Microviridae*, replacing the only recently proposed monotypic order *Petitvirales* with Microvirales would be appropriate.

Analysis of microviral diversity in terms of this new taxonomy offers new insights into the biology of this group. Unlike dsDNA and other ssDNA phages, microviruses carry no recognizable auxiliary metabolic genes or toxins involved in virulence of their bacterial hosts toward eukaryotes (e.g., see references 38, 39, and 40). Furthermore, the uptake of new genes from bacteria or other viruses is highly restricted and limited to prolific families of peptidases and methyltransferases that occur in multiple domains of life and viral realms (41, 42). Additionally, there is little evidence of frequent genetic exchange among *Microviridae* beyond the level of genus. As such, the *Microviridae* do not fall into the paradigm of widespread mosaicism that is observed in many dsDNA phages (43, 44) or eukaryotic ssDNA viruses (45). Apparently, *Microviridae* adhere to a relatively rigid genomic architecture that, due to extremely high mutation rates (46), has experienced deep exploration of its sequence space. As a result, proteins of phages with syntenic gene content can diverge beyond the thresholds generally used to denote protein families (47), leading to the establishment of highly divergent microvirus lineages with nearly identical genomic contents and organization.

The large diversity of microviruses that went unrecognized before metagenomic surveying became routine indicates a crucial role of sampling and computational analysis in their discovery. Due to their small genome sizes, sequences corresponding to microviruses are often excluded from metagenomic studies; for example, the recently published Gut Phage Database includes only those phages that are >10 kb (48). Even the conventional application of a 5-kb contig size cutoff in metagenomics excludes many members of the recently discovered Amoyvirinae or the abundant subfamily *Gokushovirinae* A. In addition to such exclusions at the computational level, many sample preparation methods, such as those employed in the Global Ocean Virome project, remove ssDNA in the extraction or library preparation steps, leading to few assemblies of microvirus genomes (29). Nonetheless, our analysis supports previous results showing that marine microvirus communities are dominated by Family 3 phages, particularly those attributed to the *Gokushovirinae* (49, 50). But notably, the genomes of marine microviruses stem from a few specialized studies that focused on marine animals (in particular, see reference 12), and almost no full microvirus genomes were recovered from large-scale global ocean studies due to their sample preparation methods.

In contrast to the exclusion of microviruses from certain samples and data sets, multiple displacement amplification methods, often used to augment samples of low DNA content, tend to enrich small, circular ssDNA molecules, yielding large amounts of *Microviridae* genomes, as in the case of the Kirstahler et al. (27) wastewater data set. This single data set contains phages from all microviral suborders and almost all putative families, perhaps not surprising in that the microviral diversity known primarily from mammalian guts is present in wastewater. The scarcity of genomes falling outside our classification scheme is encouraging and indicates that sampling of higher taxonomic units of *Microviridae* is more-or-less complete when considering the human virome. Therefore, if the human virome is well censused, the differences observed between the microvirus composition of urban and rural populations or between individuals or time points are likely to be highly accurate depictions of the dynamics of these phages.

In sum, the state of microviral taxonomy has been problematic and perhaps even an impediment to research progress. That the huge and growing diversity of microviruses of different sizes, genomic organizations, and environmental distributions have been consolidated into a single group stands in stark contrast with the plethora of taxonomic groupings afforded to dsDNA phages. Based on our analyses of thousands of microviral genomes, elevation of the *Microviridae* to a higher taxonomic rank would mitigate these problems: the order Microvirales would accommodate the diversity now known to exist within this group and assist in the taxonomic assignment of genomes recovered in metagenomic surveys, which are proving to be a continual source of microviral diversity. Overall, there is ample room for an expanded taxonomy within the viral kingdom of the *Sangervirae*, which only includes microviruses, and it would be prudent to use it.

## MATERIALS AND METHODS

### Annotation and curation of genomes.

Microvirus gene calling and host inferences were performed with PHANOTATE 1.5.0 (51) and CrisprOpenDB (52), respectively. Protein-coding genes were identified by jackhmmr searches (E value cutoff of ≤0.05) (53) using all proteins from *phi*X174, *Enterogokushovirus* EC6098, *Bdellomicrovirus phi*MH2K, Spiroplasma virus SpV4, and Chlamydia virus Chp1 (accession numbers NC_001422, NC_048874.1, NC_002643, NC_003438, and NC_001741, respectively) as the input. Conserved genes were annotated as VP1 to VP5 and VP8 in accordance with Chlamydia *virus* and *Enterogokushovirus* nomenclature. Additional genes were annotated with eggNOG-mapper version 2 (54). Genomes not containing a copy of VP1, VP4, and (with exceptions) VP2 each were discarded as incomplete.

All genomes were manually curated for quality using Geneious Prime (Biomatters Ltd.). Because all microviruses have genes facing in only one direction, we removed any gene whose orientation was the reverse of VP1. We then inspected all genomes for misannotated regions (such as multiple annotations for VP1 in a single genome) or regions that were lacking genes compared to closely related phages that were typed to the same genus (see below). For genomes from hosts using an alternative genetic code for which open reading frames were not predicted correctly, we repeated gene calling using the *Mycoplasma* code as implemented in Geneious Prime. Additionally, there were multiple instances in which MAGs were assembled in ways that split genes into multiple open reading frames through frameshifts, and these were corrected by inserting Ns into the sequences. In cases in which MAG data sets contained concatenations of two or more often identical microvirus genomes, we retained only one copy of each unique genome. Finally, all bacterial genes in contigs derived from prophages were removed and subsequently used to identify to hosts.

### Determination of family and genus membership through protein-sharing networks.

We first performed all-versus-all searches via DIAMOND 0.9.32 (55) on all microvirus proteins from genomes and prophages deposited to NCBI as of 13 April 2020 and data from Roux et al. (9) and Gregory et al. (1), as used in our previous work (18). Hits reaching an E value cutoff of 0.001 were then clustered based on having at least 80% coverage and either ≥30% and ≥50% amino acid identity (AAI) for family and genus identification, respectively. We then used the Map equation software package (http://www.mapequation.org) to sort genomes into closely related groups based on their protein content and Cytoscape 3.8.2 (56) to visualize the resulting protein-sharing networks based on the Prefuse force directed OpenCL layout. Since the vast majority of phage genomes that clustered together when applying a 50% AAI cutoff had syntenic gene contents and average pairwise nucleotide identities of ≥50% (in alignments using Clustal Omega 1.2.4, standard settings [57]), we operationally considered these phage genomes as belonging to a single genus. Membership in putative microviral families was determined via the Cytoscape network through direct connections (First Neighbor) to central VP1 proteins at ≥30% AAI. We consolidated these steps to produce the network graphs using our curated microvirus database into a pipeline termed Microvirus Organization Pipeline Using Protein sharing (MOP-UP), available at https://github.com/martinez-zacharya/MOP-UP.

### Microvirus genome detection.

To create separate alignments of VP1 proteins from each defined microviral family, we employed Clustal Omega 1.2.4, using the full distance matrix for guide tree calculation and five iterations options (57). The resulting alignments were transformed into hidden Markov models (HMMs) for use in hmmr searches, and singleton VP1 proteins and the putative capsid protein AGO48869.1 of Cellulophaga phage *phi*12a:1 (NCBI accession number KC821623) were used in jackhmmr searches with hmmer 3.2.1 (53), as described above. Searches were conducted on genomes available in the GenBank database of NCBI (as of February 2021) and all contigs available from the gut virome data set of Shkoporov et al. (4) after gene calling in PHANOTATE. Microvirus genomes were extracted, curated, and added to our database. New alignments of VP1 proteins and subsequent HMM searches were performed iteratively with the inclusion of new sequences until no new microviruses could be detected. Using the final set of HMMs from our curated database, searches for microviruses were conducted on the wastewater data set of Kirstahler et al. (27), the Cenote Human Virome Database (3), the Metagenomic Gut Virus Database (28), a global ocean virome data set of Gregory et al. (29), and three additional data sets from recent microvirus-related publications (30–32), all of which had previously undergone gene calling using PHANOTATE. Contigs containing microvirus hits were extracted and directly (i.e., without further annotation or curation for quality and completeness) used as input for MOP-UP. Family and genus membership were determined as described above.

### Phylogenetic analysis.

We extracted the VP1 and VP4 protein sequences from a randomly selected representative of each microvirus genus and created alignments with Clustal Omega 1.2.4 (57), using the full distance matrix for guide tree calculation and five iterations options. The VP1 and VP4 alignments were concatenated, and positions with >50% gaps removed using Geneious Prime (Biomatters Ltd.). We constructed phylogenies from this concatenated alignment using the WAG substitution model in FastTree 2.1.10 (58) and used Treemmer (59) to serially remove branches making the smallest contributions to tree diversity, thereby reducing the data set to 250 phages. We repeated the alignment steps with the reduced data set and estimated a phylogenetic tree with RAxML HPC (60) using the GAMMA+WAG substitution model and 100 fast-bootstrap replicates. As the resulting phylogenies were subject to low bootstrap values, we calculated transfer bootstrap estimates (TBE) (61) based on these 100 standard bootstrap repeats. Nodes with >70 TBE were collapsed using Dendroscope 3.7.5 (62). We constructed two phylogenetic trees, one for the *Microviridae* as a whole (Fig. 2B) and one confined to families 3 and 9 (Fig. 4A).

### Assessment of microviral taxon abundance in metagenomes.

SRA files from human, ocean, freshwater, soil, and ocean (3, 33, 63–65) metagenomes (Table S5) were downloaded and extracted using the NCBI SRA toolkit and processed using repair.sh and bbduk.sh (with options ktrim=r k=23 mink=11 hdist=1 qtrim=r trimq=10 minlen=100) from the BBTools package (https://sourceforge.net/projects/bbmap/). Extracted reads were mapped onto the complete MOP-UP data set using the BBTools script bbmap.sh with the option minidentity=50. The relative abundances of microviral clusters were then assessed by combining all the read numbers mapping to members of individual taxa.

### Structural analysis.

To assess homology of the putatively microviral Obscuriviridae to other phages, the putative capsid protein AGO48869.1 of Cellulophaga phage *phi*12a:1 (NCBI accession number KC821623) was submitted for structural prediction to the AlphaFold 2.1.0 Collab Server (66) in prokaryote mode using standard settings. The predicted protein structure was then submitted for structural alignments against Protein Data Bank using the Dali webserver (24).

### Data availability.

The curated database of microviruses, as well as additional microvirus and metagenomic data sets and code used for analysis, are available at https://github.com/martinez-zacharya/MOP-UP.

10.1128/mbio.00588-22.1 FIG S1 Genomic locations of accessory genes in representatives of putative microviral families. Selected genomes show the range of diversity in genome organization with regards to accessory genes within each family. For each family, representative genomes are depicted linearly starting with VP1 (dark green). Download FIG S1, EPS file, 1.7 MB. Copyright © 2022 Kirchberger et al. 2022 Kirchberger et al. https://creativecommons.org/licenses/by/4.0/ This content is distributed under the terms of the Creative Commons Attribution 4.0 International license.

10.1128/mbio.00588-22.2 FIG S2 Regions of high sequence identity denote recombination between divergent microviruses. Percent identity plots (grey) calculated for 99 nt sliding windows between the genomes of MG945336 and distantly related MG945328 (top) or closely related MG945875 (bottom). Genomes are depicted linearly starting with VP1, with the following genes noted: VP1, major capsid protein (dark green); VP2, DNA pilot protein (light green); VP4, replication initiation protein (orange); peptidases (dark blue); other/unknown genes (grey). Numbers between genes indicate percent amino acid identity between homologs. Download FIG S2, EPS file, 0.8 MB. Copyright © 2022 Kirchberger et al. 2022 Kirchberger et al. https://creativecommons.org/licenses/by/4.0/ This content is distributed under the terms of the Creative Commons Attribution 4.0 International license.

10.1128/mbio.00588-22.3 FIG S3 Identifying microviruses in metagenomic datasets. Bipartite protein-sharing networks depict groups of genomes as rectangles, connected by groups of shared proteins (triangles) at ≥30% amino acid identity. Grey rectangles and triangles represent groups of *Microviridae* genomes and proteins from the curated MOP-UP database, and red rectangles and triangles represent groups of noncurated genomes/proteins from wastewater (27). Groups containing genomes or proteins from both datasets are also colored grey. Large triangles (grey) indicate family-defining VP1 proteins, and a VP4 protein (orange triangle) links some *Microviridae* to plasmids and other mobile genetic elements (MGEs) in wastewater. Other viruses were identified by blastp searches of proteins against the NCBI database. Download FIG S3, PDF file, 1.9 MB. Copyright © 2022 Kirchberger et al. 2022 Kirchberger et al. https://creativecommons.org/licenses/by/4.0/ This content is distributed under the terms of the Creative Commons Attribution 4.0 International license.

10.1128/mbio.00588-22.4 TABLE S1 Curated microvirus genomes. Download Table S1, XLSX file, 0.2 MB. Copyright © 2022 Kirchberger et al. 2022 Kirchberger et al. https://creativecommons.org/licenses/by/4.0/ This content is distributed under the terms of the Creative Commons Attribution 4.0 International license.

10.1128/mbio.00588-22.5 TABLE S2 Protein clusters (>30% AAI) shared between putative families. Download Table S2, XLSX file, 0.03 MB. Copyright © 2022 Kirchberger et al. 2022 Kirchberger et al. https://creativecommons.org/licenses/by/4.0/ This content is distributed under the terms of the Creative Commons Attribution 4.0 International license.

10.1128/mbio.00588-22.6 TABLE S3 Microvirus genomes from additional datasets. Download Table S3, XLSX file, 0.2 MB. Copyright © 2022 Kirchberger et al. 2022 Kirchberger et al. https://creativecommons.org/licenses/by/4.0/ This content is distributed under the terms of the Creative Commons Attribution 4.0 International license.

10.1128/mbio.00588-22.7 TABLE S4 Suggested taxonomy of microviruses. Download Table S4, XLSX file, 0.01 MB. Copyright © 2022 Kirchberger et al. 2022 Kirchberger et al. https://creativecommons.org/licenses/by/4.0/ This content is distributed under the terms of the Creative Commons Attribution 4.0 International license.

10.1128/mbio.00588-22.8 TABLE S5 Metagenomes investigated in this study. Download Table S5, XLSX file, 0.01 MB. Copyright © 2022 Kirchberger et al. 2022 Kirchberger et al. https://creativecommons.org/licenses/by/4.0/ This content is distributed under the terms of the Creative Commons Attribution 4.0 International license.
