# Supplementary figures and images for: Organizing the Global Diversity of Microviruses
Source: mBio. 2022 May 2;13(3):e00588-22. doi: 10.1128/mbio.00588-22 (PMC9239249; doi:10.1128/mbio.00588-22)

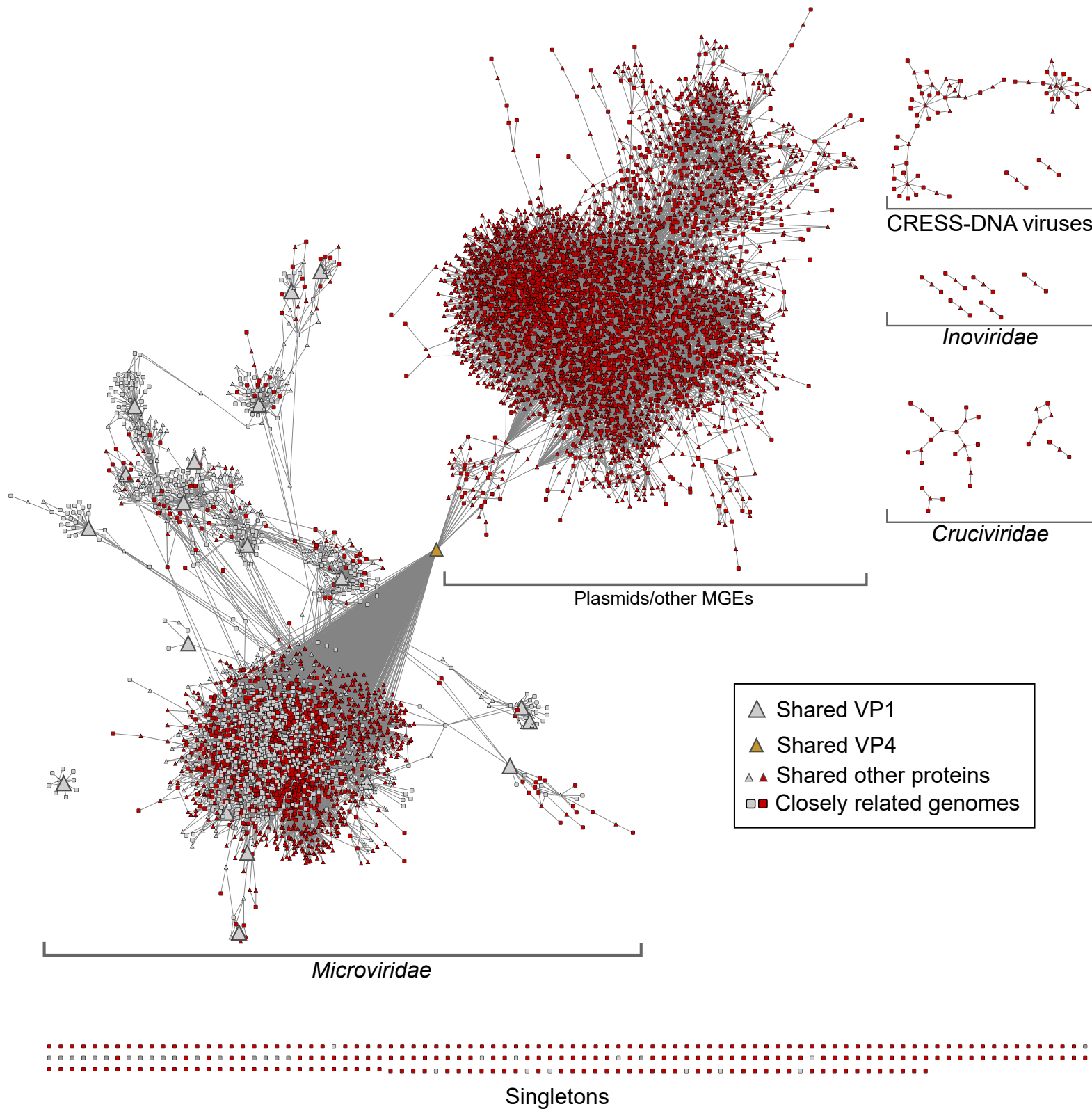

Supplement: FIG S3 [file mbio.00588-22-sf003.pdf]
